# Colour-specific diet specialization is associated with differences in owlet weight in a polymorphic owl: influence of the trophic quality variation

**DOI:** 10.1007/s00442-023-05460-4

**Published:** 2023-10-10

**Authors:** Jesús Miguel Avilés, Ángel Cruz-Miralles, Deseada Parejo

**Affiliations:** 1 Departamento de Ecología Funcional y Evolutiva, EEZA-CSIC, La Cañada de San Urbano, Almería Spain; 2 Unidad Asociada (CSIC): Ecología en el Antropoceno, Facultad de Ciencias, Universidad de Extremadura, Badajoz, Spain; 3 Departamento de Anatomía, Biología Celular y Zoología, Facultad de Ciencias, Universidad de Extremadura, Badajoz, Spain

**Keywords:** Colour polymorphism, Diet segregation, Insectivorous, Owls, Predator–prey interaction, Trophic ecology

## Abstract

The niche divergence hypothesis proposes that the evolution and maintenance of colour polymorphism is based on a mechanism of disruptive selection. In a trophic context, the hypothesis predicts that individuals differing in colour vary in their trophic niche, either because they differ in foraging efficiency or feed in different habitats. A major evolutionary conundrum is how these expectations are affected by variation in trophic quality. Using an owl species with colour plumage polymorphism, the Eurasian scops owl *Otus scops*, we examined diet and habitat segregation during reproduction in relation to plumage colouration and trophic quality. Intensive sampling revealed that trophic quality for scops owls (i.e. abundance of grasshoppers and locusts) varied more among territories than between years, but scops owls did not segregate among territories of different quality by their colouration. However, we found that sex, plumage colouration and territory differences in trophic quality explained differences in the degree of dietary specialization. Brownish males delivered a higher diversity of prey to the nest than greyish ones in high trophic quality territories. We also found that the more diverse the diet provided by males, the heavier the owlets at fledging. Our study provides evidence for a different sensitivity to trophic quality of the colour morphs with potential fitness consequences in scops owls. We highlight the importance of studying the mechanisms leading to the persistence of colour polymorphism in patchy environments, since segregation may pass otherwise unnoticed if only habitats or years with similar conditions are considered.

**Supplementary Information:**

The online version contains supplementary material available at 10.1007/s00442-023-05460-4.

## Introduction

Colour polymorphism (i.e. the occurrence of two or more phenotypically distinct, genetically based colour forms or morphs within a natural breeding population (sensu Huxley 1955)) is widespread in plants (Irwin et al. 2003; Menzies et al. 2016) and several animal taxa including invertebrates and vertebrates (Bond 2007; McLean and Stuart-Fox 2014). Its study constitutes a cornerstone of research plans that seek to understand the role of natural and sexual selection since the nineteenth century, and, in particular, an ideal study model to test how colour variants (i.e. morphs) are maintained in species subjected to natural selection and genetic drift (Darwin 1859; Ford 1945, 1957; Huxley 1955; Bond 2007; McKinnon and Pierotti 2010; McLean and Stuart-Fox 2014; White and Kemp 2016). In birds, colour polymorphism reaches particularly high incidence, being reported in 61% of avian orders and 37% of avian families (Roulin 2004b), but reaches up to 33.5% of species in the Strigiformes (i.e. owls and nightjars) (Galeotti et al. 2003). Not surprisingly, birds have been the target of a large body of empirical and comparative work aiming to understand the persistence of colour polymorphism in the wild (e.g. Fowlie and Kruger 2003; Galeotti et al. 2003; Galeotti and Rubolini 2004; Roulin 2004b; Tate et al. 2016; Passarotto et al. 2018).

A likely explanation for avian colour polymorphism is the niche divergence hypothesis based on a mechanism of disruptive selection. This hypothesis states that species occupying heterogeneous environments will benefit by exhibiting different morphs because in that way, the different colour variants will better locally adapt to different ecological niches (reviewed in Galeotti and Rubolini 2004; Roulin 2004b). This mechanism fits with recent comparative findings on owls showing that species living under more variable luminal conditions are more likely colour polymorphic (Passarotto et al. 2018). Strongest support for the mechanistic basis of the hypothesis comes from studies showing how fluctuations in luminal conditions or in colour of the habitat backgrounds can promote differences in detectability and performance of colour variants. For instance, Tate et al. (2016) found that in the white-dark polymorphic black sparrowhawk *Accipiter melanoleucus*, prey-provisioning rates differed between the morphs depending on light condition. Dark morphs delivered more prey in lower light conditions, while white morphs provisioned more in brighter conditions (Tate et al. 2016). Also, dark morphs preferred enclosed (i.e. darker) habitats to forage compared to white ones (Tate and Amar 2017), likely because in that way they can maximize concealment from prey (but see Nebel et al. 2019). Colour-dependent habitat matching and breeding performance have been also found in female, but not male, barn owls *Tyto alba* (Dreiss et al. 2012).

Temporal changes in luminosity may also promote colour polymorphism. For instance, in barn owls, moonlight variation triggered differences in vole capture success by white and dark phenotypes due to different prey reactions toward owl colour variants (San-Jose et al. 2019). In the same line, the immune response of owlets of brown and grey scops owls *Otus scops* are differently affected by moonlight variation (Avilés et al. 2022a). Beyond moonlight variation, day-night changes in luminosity may contribute to polymorphism in scops owls, as brown and grey morphs differ in their nocturnal foraging activity (Parejo et al. 2023). Also, temporal changes in the snowy landscapes may have promoted polymorphism in Fennoscandian tawny owls *Strix aluco* as the grey and the brown morph achieve different camouflage through plumage colour which may provide protection against mobbers and predators (Koskenpato et al. 2020). Hence, empirical evidence suggests that detectability of different colour morphs under variable luminal conditions or contrasting habitat backgrounds is a key driver in the maintenance of plumage colour polymorphism via disruptive selection.

In heterogeneous environments, the coexistence of colour morphs might result from differences in the trophic niche, either, because colour morphs differ in foraging efficiency of different prey, or because they segregate feeding on different micro-habitats. Evidence for trophic colour polymorphism has been reported in many taxa, including insects (Karpestam and Forsman 2011), fishes (Adams et al. 1998; Craig and Foote 2001), amphibians (Anthony et al. 2008), reptiles (Lattanzio and Miles 2016; Scali et al. 2016) or mammals (Reimchen and Klinka 2017), and, interpreted as a premise for the persistence of the polymorphism, or, a necessary prelude of on-going speciation processes (McLean and Stuart-Fox 2014). In birds, Roulin (2004a) examined this possibility in the Barn owl and found that, in Switzerland, reddish individuals fed primarily upon common voles *Microtus arvalis* whereas lighter individuals primarily upon wood mice *Apodemus spp.* Also supporting trophic segregation, Karell et al. (2021) found that in tawny owls, brown fathers provided proportionally fewer mammalian prey than grey in poor, but not in favourable mammal prey years, suggesting that the brown morph is more generalist and reacts more strongly to variation in food supply than the grey one. However, there is still limited information about the role that spatio-temporal variation in trophic quality may have played in the maintenance of colour polymorphic species, particularly for those species relying strongly on invertebrate prey, which are likely to vary relatively more spatially (i.e. among breeding territories within populations) than temporally (i.e. between years).

Colour polymorphism in the insectivorous European scops owl provides an ideal system to investigate the relationships between trophic segregation, colour and spatio-temporal variation in trophic resources. In the south of Spain, scops owls can be classed regarding plumage colour into three morphs which are equally abundant, and, whose distribution does not change between the two sexes nor across years (Parejo et al. 2018b). Pigment analyses have shown that plumage colour varies continuously from grey to brown in relation to the amount of phaeomelanin (Avilés et al. 2020). In this population, grasshoppers and locusts, are the main prey of scops owls during the breeding period (Cruz-Miralles 2021).

Here, in a first stage we study spatio-temporal variation in trophic quality for scops owls. To achieve this goal we first identified food delivered to their owlets by male and female scops owls and then sampled the availability of these food items in the territories they can occupy during four breeding seasons. In a second stage, we analysed whether diet specialization differs in relation to plumage colouration and variation in trophic quality of territories. Plumage colouration might be linked to diet specialization through its influence on behaviour. Specifically, melanin-based colourations associate with explorative behaviour and dispersal propensity in different systems (reviewed in Ducrest et al. 2008). For example, siskins *Carduelis spinus* with larger black bibs showed shorter approach latencies to a novel object (Mateos-Gonzalez and Senar 2012), and, in barn owls, darker red pheomelanic individuals moved further than paler individuals (van den Brink et al. 2012). Also, in tawny owls melanin is linked to dispersal although in interaction with environmental conditions (Passarotto et al. 2022). Given well established associations between melanins and exploratory behaviour, and recent findings showing that the exploratory profile may covary with foraging behaviour (Ersoy et al. 2022), and, ultimately influence individual diet specialization in different systems (Gharnit et al. 2022), we predict that the more pheomelanic brownish individuals will provide their offspring with a more generalized diet and higher prey diversity than grey ones. In order to identify the ultimate reason for colour trophic specialization, we also analyse the spatial segregation of the morphs in relation to trophic quality. Non-random distribution of morphs with respect to variation in territory trophic quality would be indicative that trophic segregation arose through habitat selection. Finally, aiming to understand the fitness consequences of changes in the diet for scops owls, we will examine whether differences in the diet delivered at the nests by males irrespective of their colouration explained differences in owlet condition.

## Materials and methods

### Study area

Our study was performed in the surroundings of Sierra de Baza, Granada, southeast Spain (37°18ʹ N, 3°11ʹ W). The area is an extensive agricultural landscape of around 22 km^2^ with a characteristic low density of natural holes (Avilés 2019) where 20–35 breeding pairs of scops owl are restricted to breed in cork-made nest-boxes attached to holm oak trees *Quercus ilex* (Rodríguez et al. 2011; Parejo et al. 2012). The density of scops owls in our study area averages 1.24 pairs/km^2^.

### Study species

The scops owl is a medium-sized nocturnal and trans-Saharan migrant owl arriving into the study area in April and starting its reproduction throughout May (Parejo et al. 2012; Avilés et al. 2022b). Once settled males are territorial and pairs make one clutch of about 2–6 eggs that are laid every second day. Females start incubating after laying the second egg, incubation takes 24–25 days, and nestling rearing lasts 21–29 days (Holt et al. 2021). Parental tasks are differentiated, the smaller males are in charge of courting the females prior to laying the eggs and bringing most of the food to the nest during incubation and brooding (average (SE) delivery rate is 6.9 (0.74) feeds per hour, and does not differ in relation to paternal colour (Avilés et al. 2022a), whereas the larger females incubate and guard the nest (Holt et al. 2021).

### Sampling procedure of owls

Starting in the last week of April, all nest-boxes are regularly visited once a week until egg-laying is detected. After detection of a breeding attempt, nests are visited once more just before the estimated hatching date to capture the incubating female by hand (Parejo et al. 2018b). Upon capture, females are marked with a white Tippex spot on the head that will allow their identification in video recordings. After owlet hatching, nests are visited once a week and at the end of the nesting period, we record the number of fledglings and fledgling weight to the nearest 0.50 g with a Pesola spring balance. Moreover, when the youngest chick of each nest was 18 days-old, all owlets were injected subcutaneously with phytohemagglutinin-P (PHA-P; Sigma Chemical) in the wing web to evaluate the in vivo T cell-mediated immune response replicating previously described protocols (Avilés and Parejo 2013; Avilés et al. 2022a). Adult males are captured at night with nest-traps while they bring food to nests. All individuals are metal-ringed, adults sexed based on inspection of the brood patch (only present in females (Cramp 1998)), and photographed for colour scoring. The plumage colouration of adults was scored in the head, breast and back part yielding a seven-colour scale ranging (from 3 (grey) to 9 (brown)). We have previously shown that this method of assessing plumage colour variation is repeatable across observers, and that assigned scores positively correlate with the degree of body redness measured with objective spectrophotometry (Parejo et al. 2018b).

### Nestlings diet

To study the diet we video recorded parental provisioning in 68 nests at day 8^th^ after hatching of the first egg during 2015–2018. We filmed about one hour of prey provisioning (average recording duration per nest (Range) 73.30 min (57–103)) after sunset (the time of maximum feeding activity (authors, unpublished data)), using infrared cameras (KPC-S500, black and white CCD camera, Esentia Systems Inc.) settled inside the nest-box to allow identification of delivered prey.

Based on visualization of video recordings we identified prey delivered by both parents (see Table S1 Appendix). Diet composition was estimated based on the frequency of each identified prey relative to the total number of identified prey and by the percentage of biomass the prey represents relative to the total biomass consumed. Average weights of prey used for estimating consumed biomass were extracted from previous studies carried out in the Mediterranean region (see weights and source references in Table S1 Appendix). Based on the frequency of the five more abundant taxa (i.e. *Acrididae*, *Lepidoptera*, *Araneae*, *Scolopendromorpha* and *Phasmida*, Table S1 Appendix), we calculated the Shannon diversity index which takes into account the abundance and evenness of prey types. The Shannon diversity index, however, does not reflect the type of prey and hence we calculated the proportion of the mass of *Acrididae* prey, which is the most abundant prey item, relative to all prey types.

### Trophic quality variation of territories

We measured trophic quality in all scops owl territories throughout the four years of study (*N* = 98). In addition, we measured trophic quality in potential territories occupied by Little owls *Athene noctua* (*N* = 40) and European rollers *Coracias garrulus* (*N* = 18), which also breed in the area and compete with scops owls by access to nest-boxes (Parejo et al. 2018a), and in a number of potential territories not occupied but supplied with nest-boxes (*N* = 102). In whole, we estimated spatio-temporal variation in trophic quality by performing 542 linear transects in 156 territories during the four years.

Transects were carried out from the first week of May to the last week of July (average (± SD) date: Jun 15^th^ (± 24.7 days)), which covers most of the period in which the owls are territorial in our study area (authors unpublished data). Transects were carried out from 9:00 h to 24:00 and we annotated date and time of day in order to capture the possible temporal and daily variation in trophic availability. The average number of transects per territory was variable (average (± SD) transects per territory: 4.10 (± 2.03), range: 1–9) due to the logistical impossibility of obtaining repeated samples in so many territories during a period of three months and at different times of the day. Nonetheless, still in 84% of territories transects were repeated on different dates and years, which made it possible to analyse whether trophic quality is patched in the study area.

The observer walked 50 m departing from the nest-box in a straight line at a speed of 2 km per hour, noting the number of immobile prey on the ground or in flight within a 5 m band. Invertebrates were classed into six categories: grasshoppers and locusts, butterflies and months, bumblebees, wasps, beetles and dragonflies. Grasshoppers and locusts were by far the most abundant prey category (64.8% of prey), and their numbers were strongly positively correlated with the total number of insect prey, and with prey richness both across all transects and owl breeding territories (Figure S1 appendix), indicating that the different components of trophic quality are tightly related. Therefore, we will use the number of grasshoppers and locusts reported at each territory as a proxy of trophic quality revealing both abundance and diversity of prey for scops owls. Spiders´ abundance could not be detected using the described census methodology. However, they represent only a small fraction of delivered prey for owlets (see Table S1 Appendix).

### Statistical analyses

Analyses were performed using SAS v.9.4 statistical software (SAS Institute, Cary, NC, USA).

Firstly, aiming to determine the sources of variation in trophic quality we fitted a Linear Mixed Model (LMM hereafter) on number of grasshoppers and locusts counted in the territory (distribution: Normal; link function: identity; GLIMMIX procedure in SAS) as dependent variable. The model included the year and the territory as two random intercepts and the interaction between the two to test whether differences in trophic quality among the territories hold constant over years. The model also included date of sampling as a covariate and time of day as a fixed categorical factor with five categories (morning: from 9:00 h to 12:00 h; noon: from 12:00 h to 14:00 h; siesta: from 14:00 h to 17:00 h; afternoon: from 17:00 h to 20:45 h; and night: from 20:45 to 24:00) to control, respectively, for possible seasonal and daily changes in trophic quality.

In a second step, we aimed to determine whether the diet delivered at the nests was affected by parental colouration and trophic quality at the breeding territories. We thus fitted three LMMs on diet features (i.e. prey diversity, total biomass and proportion of *Acrididae* prey) (distribution: Normal; link function: identity; GLIMMIX procedure in SAS) as dependent variables. These models included parental sex as a fixed term, and the plumage colour score of the parent (as a colour score ranging from 3 to 9) and trophic quality (estimated by the density of grasshoppers and locusts in the territory) as covariates. We also fitted the two-way interactions colour*sex and colour* trophic quality, and the three way interaction colour*sex* trophic quality. In addition, year was entered as a fixed term, and territory ID was entered as a random intercept. Finally, we entered brood size and recording duration as two further covariates to control for possible nest differences in feeding requirements and length of video-recordings possibly affecting the delivered number of feedings, respectively. After controlling for the fixed effects, the models on total biomass and proportion of *Acrididae* prey converged to a solution where the random term territory ID had not associated variation. Therefore, the random term was removed to obtain a definitive positive Hessian matrix (Kiernan et al. 2012).

In a third stage, we analysed whether scops owls segregated among territories differing in trophic quality by plumage colouration. We fitted two LMMs on plumage colouration of males and females (distribution: Normal; link function: identity; GLIMMIX procedure in SAS) as dependent variables. These models included trophic quality of the territories as covariates. In addition, nest brood size was included as a covariate to control for differences in the quality of territories not related to food. Year was entered as a random intercept to account for non-independence of measures collected over different years, but removed from the model on males because there is no variation for it after controlling for the fixed effects.

Finally, as males are the sex that provisions the most during the early nestling period (see results), we examined whether differences in diet diversity delivered by males explained differences in owlet condition. We fitted two LMMs on body mass at fledging and PHA response (distribution: Normal; link function: identity; GLIMMIX procedure in SAS) as dependent variables. These models included male and female colouration, trophic quality of the territories and diversity in diet as covariates. In addition, brood size was included as a covariate to control for possible nest differences in feeding requirements that may affect diet. Scops owl chicks hatch asynchronically so that chicks from the same nest may have slightly different ages, thus we retain owlet body mass as a predictor in the model assessing the influence of diet diversity on PHA response. Year and Nest_ID were entered as two random intercepts to account for non-independence of owlets measures collected over different years or nests. As prey diversity delivered at the nests could relate to the number of prey items brought to the nest, we also explored the association between prey diversity and total biomass delivered by males in another LMM with diet diversity as dependent variable and total biomass delivered by males as the only predictor. Year and Nest_ID were included as random intercepts in this model. Prior to fitting the models, continuous predictors were standardized to improve interpretability (Schielzeth 2010). Standard model validation graphs (Zuur 2009) revealed that model assumptions of homogeneity of variance and normality of residuals were fulfilled. *P* values smaller than 0.05 were considered significant.

## Results

### Variation in trophic quality

**Fig. 1:**
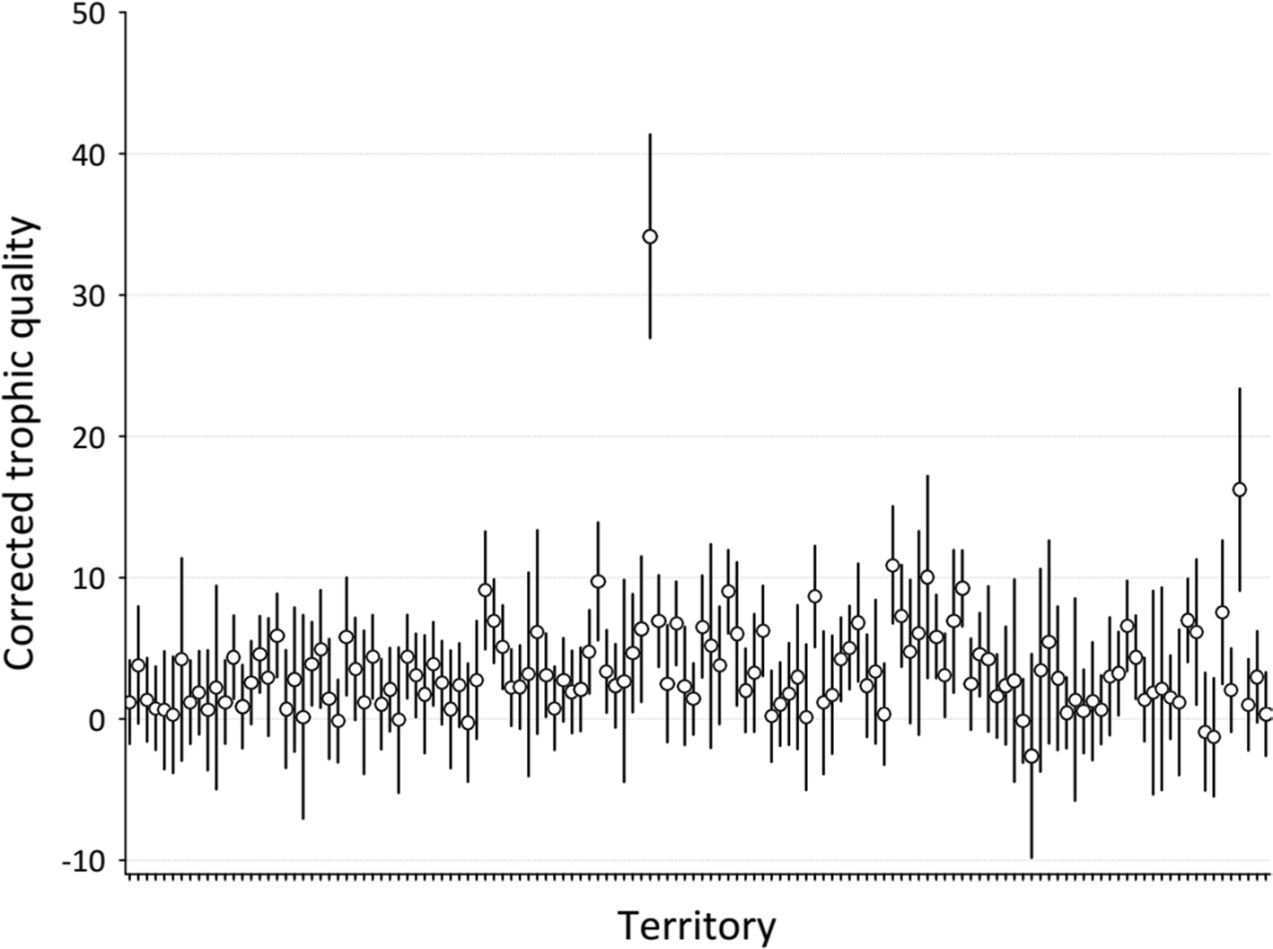
Trophic quality variation in scops owl territories (*N* = 132 territories). Average trophic quality per territory is calculated from predicted values from a GLMM analysing variation in number of grasshoppers and locusts in 542 transects in relation to year and territory as two random intercepts, and date of sampling and time of day as covariates (see Table S1 in appendix). Vertical lines are 95% confidence intervals. The conclusion that trophic quality is patchy holds when we removed the apparent outlier value of territory 161. Tick marks at the x axes correspond with territories

The number of grasshoppers and locusts counted in the transects did not differ among the four years of study (random effect of Year, Wald *Z-*test = 0.74, *P* = 0.23, Table S2 Appendix), but differed among the territories (random effect of territory, Wald *Z-*test = 3.85, *P* < 0.0001, Table S2 Appendix, (Fig. 1)). In addition, differences in grasshoppers and locusts among territories did not change with the year (random interaction effect territory*year, Wald *Z-*test = 0.76, *P* = 0.22, Table S2 Appendix).

### Diet description

We registered 2755 feeding events during the four years, but identified only a third of delivered prey (960 prey, 34.9% of feeds), due to parents entering the nest or feeding in a way that prey was not visible. Prey belonged to 16 taxa (from eight classes and twelve orders) (Table S1 Appendix). Insects constituted 86.9% of prey and 64.3% of biomass delivered, with *Orthoptera* of the family *Acrididae* (69.7%) the prey contributing the most to the biomass in the delivered prey (Table S1 Appendix). *Lepidoptera* was the second most delivered prey (11.9%), followed by *Scolopendromorpha*, *Phasmida* and *Araneae* (Table S1 Appendix). The rest of invertebrates constituted less than 2.0% of mass delivered, and vertebrates were barely represented, with reptiles (0.6%) the most abundant prey (Table S1 Appendix).

Prey-provisioning behaviour and diet delivered at the nest differed between sexes in the 2734 feeding events in which we could assign the sex (Table 1): male scops owls provided 74.8% of feeds, and provided their offspring with a more diverse diet, triple the biomass and a larger proportion of *Acrididae* than females (Table S1, Tables S3–6 Appendix, Figure S2 Appendix).

**Table 1:** Sources of variation in prey delivery to offspring in 68 scops owl nests

	Model output ‘colour score*sex*trophic quality’ interaction term			Model output ‘colour score’ explanatory variable			Model output ‘sex’ explanatory variable			Model output ‘trophic quality’ explanatory variable		
Dependent variable	DF	*F*	*P*	DF	*F*	*P*	DF	*F*	*P*	DF	*F*	*P*
Prey diversity	**1,117.2**	**5.04**	**0.027**	1,101.4	0.39	0.532	**1,64.19**	**7.97**	**0.006**	1,60.99	0.19	0.662
Total biomass	1,117.2	0.00	0.961	1,117.2	0.86	0.355	**1,117**	**27.27**	** < .0001**	1,117.1	0.79	0.377
% of *Acrididae*	1,118.6	1.47	0.227	1,118.6	1.37	0.245	**1,117.5**	**19.74**	** < .0001**	1,118.1	1.05	0.301

Model outputs of Linear mixed models analyzing the effect of sex, plumage colouration, trophic quality and their triple interaction on diet delivered at the nests by scops owls (i.e. prey diversity, total biomass, % of Acrididae). Numerator and denominator degrees of freedom are separated by point and given in the DF columns. See Tables S3–5 in the appendix for full model outputs

Significant sources of variation for each dependent variable are shown in bold

### Effect of sex, parental plumage colour and trophic quality on diet

**Fig. 2:**
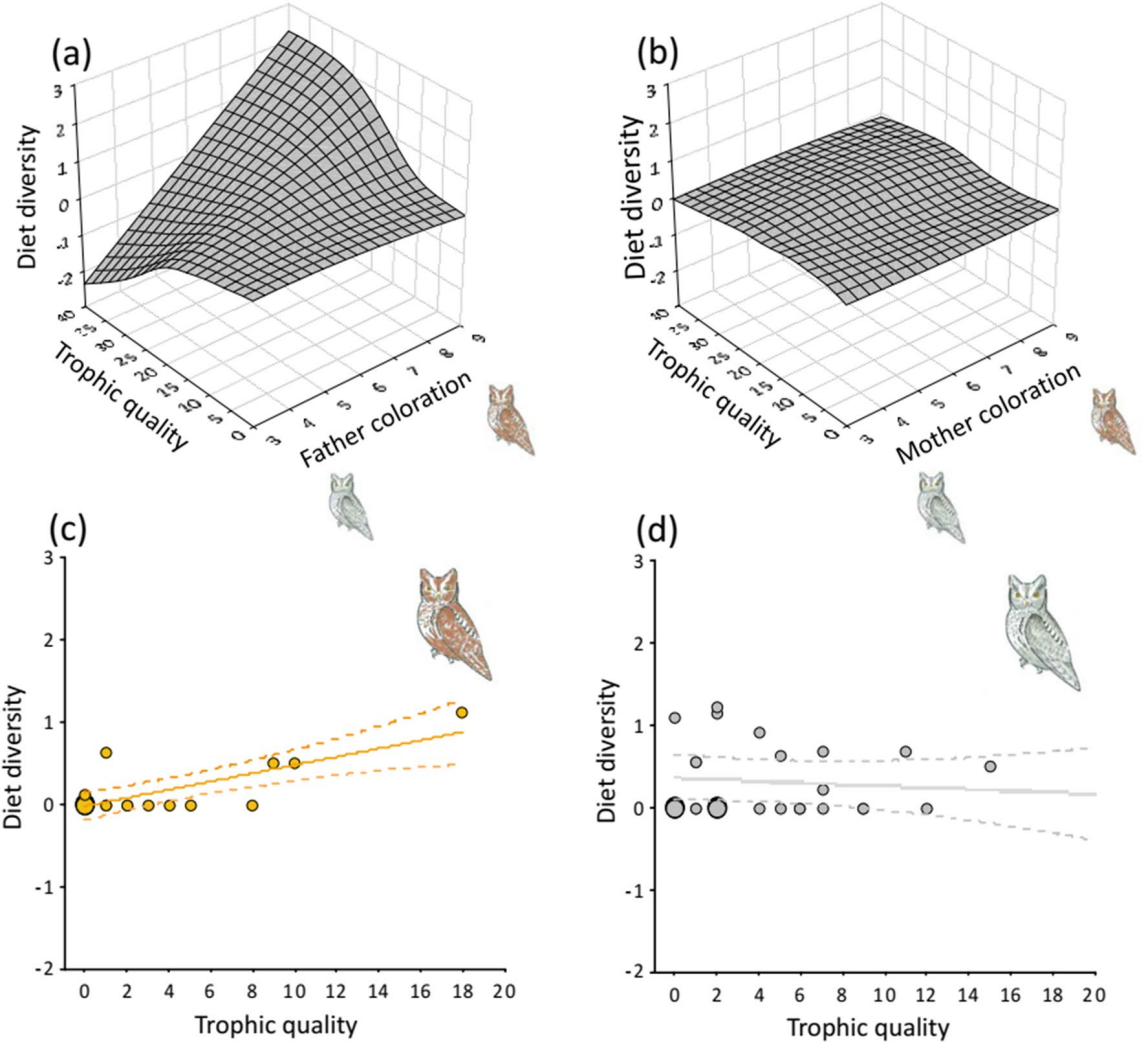
Prey diversity delivered at the nests depends on trophic quality at the territories, parental plumage colouration and sex in scops owls. **a** and **b** panels show predicted surfaces from two normal LMMs on diet diversity testing the interaction between trophic quality of the territory and plumage colouration of father and mother, respectively. **c** and **d** panels show detailed effects of trophic quality on diet diversity provided by male scops owls. Values shown are the predicted values of diversity, regression lines (continuous and dashed lines reflect significant and non-significant associations, respectively) and 95% confidence interval, for male scops owls above the third quantile (the brownest owls, *N* = 14 males) and below the first quantile (the greyest owls, *N* = 26 males) of colour variation

Prey diversity delivered at the nests was influenced by the interaction between parental plumage colour, sex and trophic quality of the breeding territory (Table S1: *F*_*1,117.2*_ = 5.04, *P* = 0.03, Table S3 appendix). We found a higher diversity of prey delivered to the nest by brownish males compared to greyer ones in territories with high trophic quality, whereas brownish and greyish males provided similar diversity in diet in territories with low trophic quality (Fig. 2A). The diversity of the diet provided by the mothers, however, was comparatively low (Fig. 2A,B and Figure S2 appendix), and invariable in relation to colouration of their plumage and the trophic quality of the territories they occupied (Fig. 2B).

More detailed analyses on males confirmed colour specific sensitivity to variation in trophic quality: diet diversity delivered at the nests by the brownest fathers increased with the trophic quality of breeding territory (*β* = 0.74, SE = 0.18, *F*_1,13_ = 16.35, *P* < 0.01, Fig. 2C), whereas the greyest fathers do not change the diversity of the diet they bring to the nests depending on the trophic quality of the territories (*β* = − 0.16, SE = 0.19, *F*_1,25_ = 0.69, *P* < 0.41, Fig. 2D).

Neither total biomass nor the proportion of *Acrididae* delivered in the nests of scops owls depended on the interaction between plumage colour, sex and trophic quality (Table S1, Table S4–5 appendix).

### Habitat distribution with respect to parental plumage colour

Variation in plumage colour of males (*F*_1,6.57_ = 6.02, *P* = 0.046, Table S6 appendix) and females (*F*_1,6.57_ = 6.02, *P* = 0.046, Table S6 appendix) did not significantly associate with trophic quality of territories (trophic quality effect, males: *F*_1,58_ = 0.02, *P* = 0.88; females: *F*_1,59.77_ = 1.18, *P* = 0.28, Table S6 appendix), indicating that scops owls differing in plumage colouration do not spatially segregate by trophic quality.

### Effect of diet quality on owlets´ condition

**Fig. 3:**
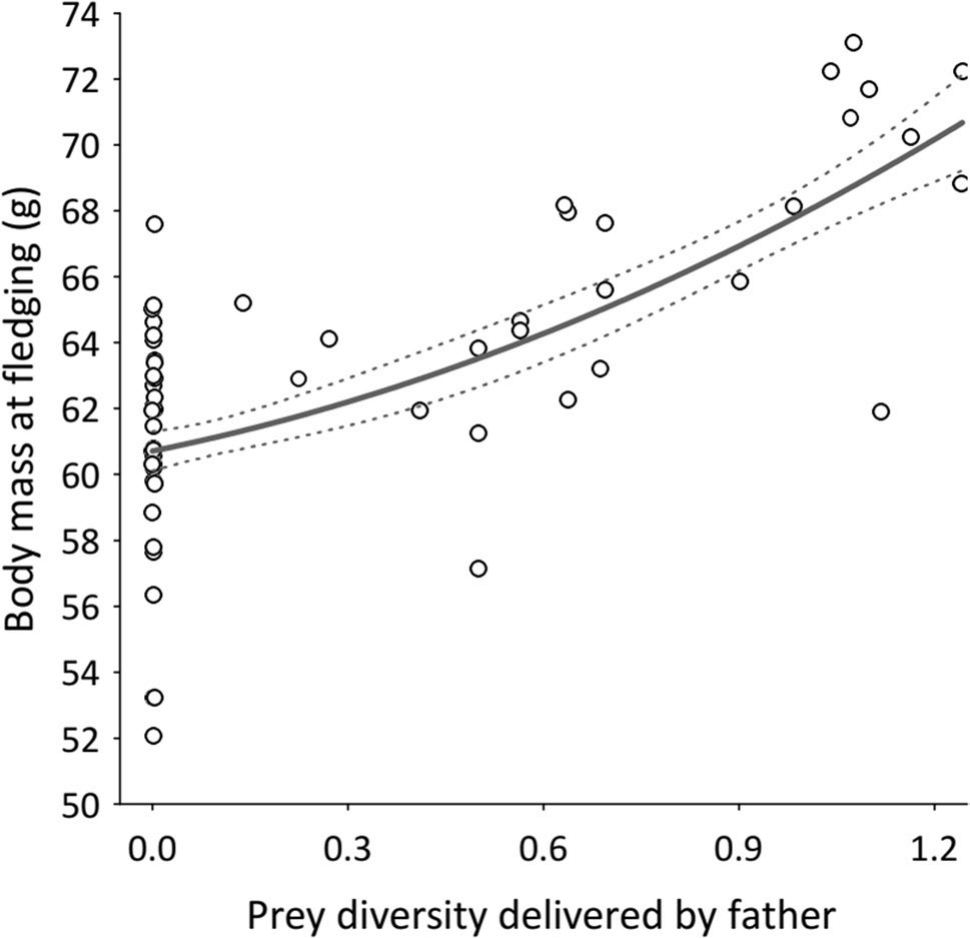
Owlet condition in relation to prey delivered by male scops owls. Predicted body mass at fledging of owlets in relation to diversity of prey delivered by fathers in scops owl nests. Data points are predicted values for owlets from the model on body mass at fledging in the Table S6 of the appendix. The bold-black line show the best polynomic adjustment of the singular relationships between body mass at fledgling and prey delivered, and the 95% CLs are represented by corresponding dashed lines. *N* = 138 owlets in 57 nests

Fledging body mass of owlets was influenced by diet diversity delivered at the nests (Table S7 appendix, *F*_1,6.57_ = 6.02, *P* = 0.046). Body mass of owlets increased when males delivered a more diverse diet (Fig. 3). We also found that males providing a more diverse diet delivered more prey at their nests (LMM; total biomass effect on diet diversity: Beta (SE) 0.35 (0.08), *F*_1,228.9 _= 18.91, *P* = 0.0001). However, owlet immuno-competence, as measured by the PHA response, was not influenced by diet diversity, but by the number of fledging at the nests and the plumage colouration of the father (Table S7 and Fig. 3 in Appendix).

## Discussion

We found that individuals with different colour plumage show different sensitivity to trophic quality variation in a polymorphic owl. Brownish males delivered a higher diversity of prey for their owlets when they bred in territories with greater abundance and diversity of prey, whereas the greyest males fed their owlets with a similar diet in territories of high and low trophic quality. Hence, browner scops owl males are more generalist and sensitive to trophic quality changes than grey males. In addition, we have found that owlets fed with a more diverse diet had a higher weight at fledging, suggesting that the generalist strategy of the brown male may have adaptive value. We have also shown that colour-dependent sensitivity to trophic quality variation was not due to a non-random territory distribution of owls with different plumage colouration. Our findings illustrate a previously neglected role of the spatial variation in trophic quality in the maintenance of polymorphisms in natural populations.

Here we have shown that in the south of Spain, the scops owl shows a high dependence of insects that constituted the most important prey in number and biomass delivered in the nests (Table S1 in Appendix, Holt et al. 2021). Moreover, massive sampling of insect prey in potential breeding territories revealed that the variation in scops owls’ main prey between territories is greater than the variation between years. Hence, different territories show intrinsic differences in trophic quality for scops owls, which would be a key premise for a trophic segregation based on colouration. Current knowledge about predator–prey trophic specialization in colour polymorphic birds comes from studies carried out in northern latitudes, where predator birds specialize on small mammals (e.g. *Microtus* spp.), which exhibit marked inter-annual variation in abundance (Stenseth 1999). Indeed, Karell et al. (2021) have recently found that in years when the tawny owls’ main prey in Finland are abundant, both brown and grey morphs prey on mammals, but in years with low abundance of small mammals, the brown morph is more prone to switch to small bird predation than the grey one. Also, Roulin (2004a) studied changes in the abundance of small mammals in the diet of polymorphic barn owls in Switzerland on a yearly basis.

Although trophic quality is patchy, we have found that different coloured scops owls did not segregate among territories by trophic quality. Several mutually non-exclusive explanations to the absence of colour segregation are possible. First, the scops owl is a late breeder in the hole-nesting bird assembly breeding in our study area and unable to outcompete already settled Little owls and European rollers in their access to nest-boxes (Parejo et al. 2012; Avilés et al. 2019). Also, rollers (Parejo et al. 2013), and to a lesser extent little owls (Del Hoyo et al. 1999), prey on large insects in the south of Spain. Therefore, scops owls may not be able to occupy territories based on its trophic quality due to interference competition with rollers and little owls. Supporting this possibility, the average number of grasshoppers and locusts counted in the transects was 4.43 (95% confidence interval: 3.38–5.48, *n* = 87)) in territories hold by rollers and little owls, and 3.11 (95% confidence interval: 2.31–3.91 *n* = 153)) in the territories hold by scops owls (One-way ANOVA: *F*_1,283_ = 3.88, *P* = 0.04). Also, it could be argued that the quality of a breeding territory for the scops owls might not be exclusively determined by the abundance of main prey, but by other aspects of quality such as the avoidance of predation threats (Parejo et al. 2012), or of human disturbance by farming activities (Exposito-Granados et al. 2020). Indeed, in the polymorphic white-throated sparrow *Zonotrichia albicollis*, tan and white males settled in territories of different quality in terms of social interactions, white males tending to settle in high-density areas where the probability of encountering neighbouring fertile females is the greatest (Formica et al. 2004). Although further research is clearly needed to discriminate among these possibilities, the finding that brown and grey coloured scops owls do not occupy territories differing in trophic quality suggest that morph differences in diet specialization are likely due to hunting efficiency being linked to colour plumage, such as has been shown in colour polymorphic barn owls (San-Jose et al. 2019). We have previously found some clues of hunting efficiency linked to colour in scops owls as body mass gain of owlets in relation to moonlight depends on parental colouration (Avilés et al. 2022a), but this remains to be experimentally tested.

Our finding of brownish males being more generalistic than greyer males fits with expectations based on the role of melanins in exploratory behaviour (Ducrest et al. 2008), and agrees with previous findings in tawny owls showing that pheomelanic individuals would be more generalist (Karell et al. 2021). Although the possible mechanisms linking exploratory behaviour to trophic specialization in scops owls clearly deserve much more investigation, our results provide evidence that behaviours related to plumage colour may relate to the trophic behaviour of an owl (Roulin 2004a; Karell et al. 2021), in line with results of recent studies showing links between individual behaviour and resource use in other species or ecological contexts (Ersoy et al. 2022; Gharnit et al. 2022).

We also found that the more diverse the diet provided by male scops owls, the heavier the owlets at fledging. In many owl species, being bigger at fledging provides a fitness benefit in terms of post-fledging survival and is linked to parental colouration. For instance, in tawny owls, owlets converted food more efficiently into body mass when their biological mother was dark pheomelanic (Piault et al. 2009). Also, Morosinotto et al. (2020) showed that across 40 years of data offspring of pheomelanic parents were heavier at fledgling than offspring of light grey parents, thus potentially having a post-fledging fitness advantage. Therefore, although we do not directly test for an association between parental colouration and fitness prospect of owlets in this study (see however Avilés et al. 2022a), this result would be in line with previous findings on colour polymorphic owls suggesting fitness benefits of the pheomelanic individuals breeding in high quality territories. However, probing the fitness benefits of a diverse diet in scops owls would deserve future experimentation, as prey diversity is positively correlated with total biomass delivered by males in our population (see results). Hence, the link between diet diversity and body mass at fledging might be because males providing a more diverse diet also deliver more prey. Finally, it is worth stressing that the diversity of the diet provided by the mothers was not related to their colouration or the trophic quality of the territories they occupied, likely due to the minor role of females in feeding owlets (Figure S2 Appendix).

The different ability of coloured morphs to cope with changes in trophic quality allows us to speculate about temporal trends in the colour phenotype of the scops owls. Recent studies suggest that increasing temperature and drought under climate change might trigger the decline of native insect communities in anthropogenic Mediterranean ecosystems (Uhl et al. 2022). However, the effects of climate change are likely taxon-specific and may favour the invasion of exotic insects (Gutierrez and Ponti 2014), or be exacerbated by land-use changes (Outhwaite et al. 2022), which might induce sudden and unpredictable changes in the abundance and diversity of insect communities. In this scenario, specialist predators are expected to do worse than generalistic ones, because they are not that efficient when their preferred prey are scarce (Terraube et al. 2011; Karell et al. 2021). This predicts an increase in frequency of the brown scops owl phenotypes over time. Fitting this expectation, a longitudinal study investigating temporal trends in an index of plumage colour of Italian scops owls from museum collection showed that the frequency of the brown morph increased in Italy throughout the twentieth century (Galeotti et al. 2009). Temporal trends in scops owl colouration was partly explained by the increase in temperature and rainfall during the last century, but the mechanisms behind the patterns were not investigated (Galeotti et al. 2009). Our results would provide a potential mechanistic basis for temporal trends in scops owl colouration based on the greater ability of brown individuals to take advantage of unpredictable changes in the abundance or diversity of potential prey. However, it is critical to carry out experimental studies manipulating aspects of trophic quality for scops owls affected by climate change to identify the exact mechanism behind the apparent greater feeding efficiency of brown individuals when resources are abundant.

## Conclusions

Our study provides important supporting evidence that spatial variation in food abundance influences the trophic behaviour of a polymorphic owl with potential fitness consequences. The existence of small-scale variations in environmental conditions might promote disruptive selection processes in which different morphs coexist into populations by segregating in different environments (reviewed in Galeotti and Rubolini 2004; Roulin 2004b). Our study reveals that morph segregation into territories might be complicated by interference competition with other species using same resources but could be effective by diet specialization. Finally, we stress that studying trophic specialization in relation to colouration in southern latitudes may open new avenues in our understanding of predator–prey polymorphisms, because in these latitudes, predators can specialize in consuming invertebrate prey that present dynamics that have been little studied. Our findings are important because they allow a better understanding of the dynamic of colour polymorphisms in the current scenario of strong fluctuations of trophic quality promoted by global change.

### Supplementary Information

Below is the link to the electronic supplementary material.

Supplementary file1 Average weights of prey used for estimating consumed biomass were extracted from previous studies carried out in the Mediterranean region (see weights and source references in Table S1 Appendix). (DOCX 299 KB)

## Data Availability

Upon acceptance of the manuscript, data and scripts will be archived at the digital repository of the Spanish Council for Research (digital.csic.es).
