# Supplementary material for: Colour-specific diet specialization is associated with differences in owlet weight in a polymorphic owl: influence of the trophic quality variation
Source: Oecologia. 2023 Oct 10;203(1-2):181–91. doi: 10.1007/s00442-023-05460-4 (PMC10615958; doi:10.1007/s00442-023-05460-4)
Supplement: Supplementary file 1 — Supplementary file1 Average weights of prey used for estimating consumed biomass were extracted from previous studies carried out in the Mediterranean region (see weights and source references in Table S1 Appendix). (DOCX 299 KB) [file 442_2023_5460_MOESM1_ESM.docx]

Supplementary material for:

**Colour-specific diet specialization is associated with differences in owlet weight in a polymorphic owl: Influence of the trophic quality variation**

**Jesús Miguel Avilés^1,2^_,_ Ángel Cruz-Miralles^3^, and Deseada Parejo^1,2^**

***^1^Departamento de Ecología Funcional y Evolutiva, EEZA-CSIC, La Cañada de San Urbano, Almería, España.***

***^2^Unidad Asociada (CSIC): Ecología en el Antropoceno, Facultad de Ciencias, Universidad de Extremadura, Badajoz, España***

***^3^Departamento de Anatomía, Biología Celular y Zoología, Facultad de Ciencias, Universidad de Extremadura, Badajoz, España.***

****Corresponding author: Avilés J.M. (javiles@eeza.csic.es)***

**Table 1 Appendix. Prey delivered to offspring in the nests by male and female scops owls. The total columns also include prey that were not assigned to any sex. Data by prey class are shown in bold and were calculated from the sum of minor taxa.**

|  | **Males** | | | **Females** | | | **Total** | | |
| --- | --- | --- | --- | --- | --- | --- | --- | --- | --- |
| **Prey taxa** | **n** | **Frequency**  **(%)** | **Biomass**  **(%)** | **n** | **Frequency**  **(%)** | **Biomass**  **(%)** | **n** | **Frequency**  **(%)** | **Biomass**  **(%)** |
| **Class *Clitellata*** | **2** | **0.3** | **0.6** | **0** | **0.0** | **0.0** | **2** | **0.2** | **0.4** |
| Order *Crassiclitellata* | 2 | 0.3 | 0.6 | 0 | 0.0 | 0.0 | 2 | 0.2 | 0.4 |
| Fam. *Lumbricidae*^1^ | 2 | 0.3 | 0.6 | 0 | 0.0 | 0.0 | 2 | 0.2 | 0.4 |
| **Class *Arachnida*** | **24** | **3.3** | **4.9** | **15** | **6.4** | **6.4** | **39** | **4.1** | **5.5** |
| Order *Araneae*^2^ | 24 | 3.3 | 4.9 | 15 | 6.4 | 6.4 | 39 | 4.1 | 5.5 |
| **Class *Chilopoda*** | **35** | **4.9** | **16.3** | **27** | **11.5** | **11.5** | **63** | **6.6** | **20.5** |
| Order *Scolopendromorpha*^3^ | 35 | 4.9 | 16.3 | 27 | 11.5 | 11.5 | 63 | 6.6 | 20.5 |
| **Class *Diplopoda*** | **3** | **0.4** | **0.2** | **0** | **0.0** | **0.0** | **3** | **0.3** | **0.1** |
| Order *Julida*^4^ | 3 | 0.4 | 0.2 | 0 | 0.0 | 0.0 | 3 | 0.3 | 0.1 |
| **Class *Insecta*** | **647** | **90.0** | **70.5** | **190** | **81.2** | **81.3** | **834** | **86.9** | **64.3** |
| Order *Odonata*^5^ | 2 | 0.3 | 0.3 | 0 | 0.0 | 0.0 | 2 | 0.2 | 0.2 |
| Order *Phasmida*^6^ | 48 | 6.7 | 8.6 | 9 | 3.8 | 3.8 | 57 | 5.9 | 7.1 |
| Order *Orthoptera* | 503 | 70.0 | 58.6 | 160 | 68.4 | 68.5 | 669 | 69.7 | 54.4 |
| Fam. *Acrididae*^7^ | 497 | 69.1 | 57.9 | 157 | 67.1 | 67.2 | 660 | 68.8 | 53.6 |
| Fam. *Tettigoniidae*^8^ | 5 | 0.7 | 0.6 | 3 | 1.3 | 1.3 | 8 | 0.8 | 0.7 |
| Fam. *Gryllidae*^9^ | 1 | 0.1 | 0.1 | 0 | 0.0 | 0.0 | 1 | 0.1 | 0.1 |
| Order *Mantodea*^10^ | 1 | 0.1 | 0.3 | 0 | 0.0 | 0.0 | 1 | 0.1 | 0.2 |
| Order *Lepidoptera* | 93 | 12.9 | 2.7 | 21 | 9.0 | 9.0 | 114 | 11.9 | 2.4 |
| Imago^11^ | 90 | 12.5 | 2.6 | 21 | 9.0 | 9.0 | 111 | 11.6 | 2.3 |
| Larvae^12^ | 3 | 0.4 | 0.1 | 0 | 0.0 | 0.0 | 3 | 0.3 | 0.1 |
| **Class *Sauropsida*** | **6** | **0.8** | **4.4** | **0** | **0.0** | **0.0** | **6** | **0.6** | **3.1** |
| Order *Squamata* | 6 | 0.8 | 4.4 | 0 | 0.0 | 0.0 | 6 | 0.6 | 3.1 |
| Fam. *Gekkonidae*^13^ | 1 | 0.1 | 1.0 | 0 | 0.0 | 0.0 | 1 | 0.1 | 0.7 |
| Fam. *Lacertidae*^14^ | 3 | 0.4 | 2.2 | 0 | 0.0 | 0.0 | 3 | 0.3 | 1.6 |
| Fam. *Amphisbaenidae*^15^ | 2 | 0.3 | 1.2 | 0 | 0.0 | 0.0 | 2 | 0.2 | 0.8 |
| **Class Aves** | **0** | **0.0** | **0.0** | **1** | **0.4** | **0.4** | **1** | **0.1** | **2.9** |
| Order *Passeriformes*^16^ | 0 | 0.0 | 0.0 | 1 | 0.4 | 0.4 | 1 | 0.1 | 2.9 |
| **Class Mammalia** | **2** | **0.3** | **3.1** | **1** | **0.4** | **0.4** | **3** | **0.3** | **3.2** |
| Order *Rodentia*^17^ | 2 | 0.3 | 3.1 | 1 | 0.4 | 0.4 | 3 | 0.3 | 3.2 |

**Source for mass in grams of the different taxa:** 1: 3.2g (Daniel et al. 1996); 2: 2.08 (Moya-Larano et al. 2002); 3: 4.8g (Franco and Andrada 1977); 4: 0.5g (Franco and Andrada 1977); 5: 1.59 g (as average of *Coenagrion puella*, *Aeshna cyanea*, *Aeshna mixta* and *Sympetrum striolatum* (Clarke et al. 1996); 6: 1.85g (comparing with *Obrimus asperrimus* (Frantsevich and Cruse 1997); 7: 1.2g (Fargallo et al. 2020); 8: 1.2g (Fargallo et al. 2020); 9: 1.2g (Fargallo et al. 2020); 10: 3g (Franco and Andrada 1977); 11: 0.13g (Marchesi and Sergio 2005); 12: 0.4g (Naef-Daenzer and Keller 1999); 13: 10.05g (Zuffi et al. 2011); 14: 7.62g (Zamora-Camacho et al. 2014); 15: 6g (Franco and Andrada 1977); 16: 43g and 17 (Cramp 1998): 16g (Franco and Andrada 1977).

**Table 2 Appendix. Sources of variation in trophic quality for scops owls.** Results of the Linear Mixed Model analysing variation in the number of grasshoppers and locusts (normal error structure) across territories and years (N=542 transects in 156 territories). Significant terms (95% confidence interval does not include the null) are highlighted in bold and predictors were standardized to improve interpretability. Degrees of freedom are obtained using the Satterthwaite method.

|  |  |  | |  |  | |  | | |  | |  | | |  | | |  |
| --- | --- | --- | --- | --- | --- | --- | --- | --- | --- | --- | --- | --- | --- | --- | --- | --- | --- | --- |
|  |  |  |  |  |  |  |  |  |  |  |  |  |  |  |  |  |  |  |
|  | **Time** | **Estimate** | | **Standard error** | **DF** | | ***F*** | | | **P** | | **Lower** | | | **Upper** | | |  |
| **Fixed Effect** |  |  |  |  |  |  |  |  |  |  |  | **95%** | | | **95%** | | |  |
|  |  |  |  |  |  |  |  |  |  |  |  | **CL** | | | **CL** | | |  |
| Intercept |  | -8.04 | | 1.55 | 1,126.8 | | 5.18 | | | <0.0001 | | -11.12 | | | -4.97 | | |  |
| **Date** |  | **0.06** | | **0.007** | **1,382.0** | | **73.98** | | | **<0.0001** | | **0.05** | | | **0.08** | | |  |
| Time | afternoon | 0.98 | | 0.97 | 4,488.7 | | 1.52 | | | 0.19 | | -0.92 | | | 2.88 | | |  |
|  | noon | 1.38 | | 0.91 |  | |  | | |  | | -0.40 | | | 3.16 | | |  |
|  | morning | 1.40 | | 0.92 |  | |  | | |  | | -0.41 | | | 3.22 | | |  |
|  | night | -0.50 | | 1.32 |  | |  | | |  | | -3.10 | | | 2.10 | | |  |
|  | siesta† | 0.00 | | . |  | |  | | |  | | . | | | . | | |  |
| **Random terms** |  |  | |  |  | |  | |  | |  | |  | | |  |  |  |
|  | **Estimate** | **Standard error** | | **Wald*-Z*** | **P** | |  | |  | |  | |  | | |  |  |  |
| **Territory** | **5.49** | **1.61** | | **3.41** | **0.0003** | |  | |  | |  | |  | | |  |  |  |
| Year | 0.62 | 0.87 | | 0.72 | 0.23 | |  | |  | |  | |  | | |  |  |  |
| Territory*Year | 1.16 | 1.53 | | 0.76 | 0.22 | |  | |  | |  | |  | | |  |  |  |
| †Reference level | | |  |  | |  | |  | | |  |  | |  | | |  | |

**Table 3 Appendix.** Results of the Linear Mixed Model analysing variation in prey diversity (normal error structure). Significant terms (95% confidence interval does not include the null) are highlighted in bold and predictors were standardized to improve interpretability. Degrees of freedom are obtained using the Satterthwaite method.

| **Prey diversity** | |  | |  | |  | |  | |  |  |  |  |
| --- | --- | --- | --- | --- | --- | --- | --- | --- | --- | --- | --- | --- | --- |
| **(N=68 nests)** | |  |  |  |  |  |  |  |  |  |  |  |  |
|  | | **Sex** | | **Estimate** | | **Standard error** | | **DF** | | ***F*** | **P** | **Lower** | **Upper** |
| **Fixed Effect** | |  |  |  |  |  |  |  |  |  |  | **95%** | **95%** |
|  | |  |  |  |  |  |  |  |  |  |  | **CL** | **CL** |
| Intercept | |  | | 0.32 | | 0.15 | | 1,3.14 | | 2.22 | 0.11 | -0.13 | 0.78 |
| Brood size | |  | | 0.05 | | 0.03 | | 1,70.01 | | 2.08 | 0.154 | -0.02 | 0.11 |
| **Sex** | | Female | | -0.14 | | 0.05 | | **1,64.19** | | **7.97** | **0.006** | **-0.23** | **-0.04** |
|  | | Male† | | 0.00 | | . | |  | |  |  | . | . |
| Record duration | |  | | -0.02 | | 0.03 | | 1,60.43 | | 0.31 | 0.580 | -0.09 | 0.05 |
| Colour | |  | | 0.02 | | 0.05 | | 1,101.4 | | 0.39 | 0.532 | -0.07 | 0.11 |
| Colour*Sex | | Female | | -0.08 | | 0.06 | | 1,116.4 | | 1.56 | 0.214 | -0.20 | 0.04 |
|  | | Male† | | 0.00 | | . | |  | |  |  | . | . |
| Hatching date | |  | | -0.05 | | 0.04 | | 1,61.79 | | 2.02 | 0.161 | -0.13 | 0.02 |
| Trophic quality | |  | | 0.02 | | 0.04 | | 1,60.99 | | 0.19 | 0.662 | -0.06 | 0.09 |
| **Colour*Sex*** Trophic quality | | Female | | -0.05 | | 0.05 | | **1,117.2** | | **5.04** | **0.027** | **0.01** | **0.31** |
|  | | Male† | | 0.16 | | 0.07 | |  | |  |  |  |  |
| Colour* Trophic quality | |  | | 0.00 | | . | | 1,97.45 | | 1.79 | 0.184 | -0.14 | 0.05 |
| **Random terms** |  | |  | |  | |  | |  |  |  |  |  |
|  | **Estimate** | | **Standard error** | | **Wald*-Z*** | | **P** | |  |  |  |  |  |
| Territory ID | 0.02 | | 0.01 | | 1.81 | | 0.03 | |  |  |  |  |  |
| Year | 0.08 | | 0.07 | | 1.16 | | 0.12 | |  |  |  |  |  |

†Reference level

**Table 4 Appendix.** Results of the Linear Mixed Model analysing variation in total biomass (normal error structure). Significant terms (95% confidence interval does not include the null) are highlighted in bold and predictors were standardized to improve interpretability. Degrees of freedom are obtained using the Satterthwaite method.

| **Total biomass** | |  | |  | |  | |  | |  |  |  |  |
| --- | --- | --- | --- | --- | --- | --- | --- | --- | --- | --- | --- | --- | --- |
| **(N=68 nests)** | |  |  |  |  |  |  |  |  |  |  |  |  |
|  | | **Sex** | | **Estimate** | | **Standard error** | | **DF** | | ***F*** | **P** | **Lower** | **Upper** |
| **Fixed Effect** | |  |  |  |  |  |  |  |  |  |  | **95%** | **95%** |
|  | |  |  |  |  |  |  |  |  |  |  | **CL** | **CL** |
| Intercept | |  | | 9.61 | | 3.63 | | 1,2.65 | | 2.65 | 0.07 | -1.59 | 20.81 |
| Brood size | |  | | 0.85 | | 0.66 | | 1,117.5 | | 1.64 | 0.203 | -0.46 | 2.15 |
| **Sex** | | Female | | -6.51 | | 1.25 | | **1,117** | | **27.27** | **<.0001** | **-8.98** | **-4.04** |
|  | | Male† | | 0.00 | | . | |  | |  |  | . | . |
| Record duration | |  | | -0.37 | | 0.68 | | 1,118.5 | | 0.29 | 0.588 | -1.72 | 0.98 |
| Colour | |  | | 0.50 | | 1.10 | | 1,117.2 | | 0.86 | 0.355 | -1.68 | 2.68 |
| Colour*Sex | | Female | | -2.27 | | 1.39 | | 1,117.2 | | 2.67 | 0.105 | -5.02 | 0.48 |
|  | | Male† | | 0.00 | | . | |  | |  |  | . | . |
| Hatching date | |  | | -0.66 | | 0.75 | | 1,118.9 | | 0.77 | 0.381 | -2.16 | 0.83 |
| Trophic quality | |  | | -0.66 | | 0.74 | | 1,117.1 | | 0.79 | 0.377 | -2.13 | 0.81 |
| Colour*Sex* Trophic quality | | Female | | -1.24 | | 1.11 | | 1,117.2 | | 0.00 | 0.961 | -3.43 | 0.95 |
|  | | Male† | | -1.14 | | 1.73 | |  | |  |  | -4.57 | 2.28 |
| Colour* Trophic quality | |  | | 0.00 | | . | | 1,117.1 | | 1.40 | 0.240 | . | . |
| **Random terms** |  | |  | |  | |  | |  |  |  |  |  |
|  | **Estimate** | | **Standard error** | | **Wald*-Z*** | | **P** | |  |  |  |  |  |
| Territory ID‡ |  | |  | |  | |  | |  |  |  |  |  |
| Year | 49.49 | | 41.81 | | 1.18 | | 0.12 | |  |  |  |  |  |

†Reference level

‡Territory ID was removed from the model to obtain a definitive positive Hessian matrix.

**Table 5 Appendix.** Results of the Linear Mixed Model analysing variation in proportion of *Acrididae* prey (normal error structure). Significant terms (95% confidence interval does not include the null) are highlighted in bold and predictors were standardized to improve interpretability. Degrees of freedom are obtained using the Satterthwaite method.

| **Proportion of *Acrididae*** | |  | |  | |  | |  | |  |  |  |  |
| --- | --- | --- | --- | --- | --- | --- | --- | --- | --- | --- | --- | --- | --- |
| **(N=68 nests)** | |  |  |  |  |  |  |  |  |  |  |  |  |
|  | | **Sex** | | **Estimate** | | **Standard error** | | **DF** | | ***F*** | **P** | **Lower** | **Upper** |
| **Fixed Effect** | |  |  |  |  |  |  |  |  |  |  | **95%** | **95%** |
|  | |  |  |  |  |  |  |  |  |  |  | **CL** | **CL** |
| Intercept | |  | | 0.41 | | 0.11 | |  | |  |  | 0.12 | 0.69 |
| Brood size | |  | | -0.04 | | 0.06 | | 1,120 | | 0.39 | 0.534 | -0.15 | 0.08 |
| **Sex** | | Female | | 0.49 | | 0.11 | | **1,117.5** | | **19.74** | **<.0001** | **0.27** | **0.72** |
|  | | Male† | | 0.00 | | . | |  | |  |  | . | . |
| Record duration | |  | | -0.03 | | 0.06 | | 1,112.3 | | 0.29 | 0.594 | -0.15 | 0.09 |
| Colour | |  | | 0.03 | | 0.10 | | 1,118.6 | | 1.37 | 0.245 | -0.17 | 0.22 |
| Colour*Sex | | Female | | 0.09 | | 0.12 | | 1,119 | | 0.49 | 0.483 | -0.16 | 0.33 |
|  | | Male† | | 0.00 | | . | |  | |  |  | . | . |
| Hatching date | |  | | -0.06 | | 0.06 | | 1,102.2 | | 0.73 | 0.396 | -0.18 | 0.07 |
| Trophic quality | |  | | -0.07 | | 0.07 | | 1,118.1 | | 1.05 | 0.301 | -0.20 | 0.06 |
| Colour*Sex* Trophic quality | | Female | | -0.05 | | 0.10 | | 1,118.6 | | 1.47 | 0.227 | -0.24 | 0.14 |
|  | | Male† | | 0.17 | | 0.15 | |  | |  |  | -0.13 | 0.48 |
| Colour* Trophic quality | |  | | 0.00 | | . | | 1,117.6 | | 0.48 | 0.491 | . | . |
| **Random terms** |  | |  | |  | |  | |  |  |  |  |  |
|  | **Estimate** | | **Standard error** | | **Wald*-Z*** | | **P** | |  |  |  |  |  |
| Territory ID‡ |  | |  | |  | |  | |  |  |  |  |  |
| Year | 0.02 | | 0.03 | | 0.81 | | 0.21 | |  |  |  |  |  |

†Reference level

‡Territory ID was removed from the model to obtain a definitive positive Hessian matrix.

**Table 6 Appendix.** Results of the Linear Mixed Models analysing the association between male and female plumage coloration (normal error structure) and trophic quality (number of grasshoppers and locusts). Significant terms (95% confidence interval does not include the null) are highlighted in bold and predictors were standardized to improve interpretability. Degrees of freedom are obtained using the Satterthwaite method.

| **Male plumage coloration** |  | | |  |  | |  | |  | |  |  |
| --- | --- | --- | --- | --- | --- | --- | --- | --- | --- | --- | --- | --- |
| **(N=61)** |  |  |  |  |  |  |  |  |  |  |  |  |
|  | **Estimate** | | | **Standard error** | **DF** | | ***F*** | | **P** | | **Lower** | **Upper** |
| **Fixed Effect** |  |  |  |  |  |  |  |  |  |  | **95%** | **95%** |
|  |  |  |  |  |  |  |  |  |  |  | **CL** | **CL** |
| Intercept | -0.37 | | | 0.45 | 1,58 | | -0.84 | | 0.407 | | -1.27 | 0.52 |
| Brood size | 0.07 | | | 0.11 | 1,58 | | 0.44 | | 0.509 | | -0.15 | 0.30 |
| Trophic quality | -0.02 | | | 0.14 | 1,58 | | 0.02 | | 0.88 | | -0.29 | 0.25 |
| **Random term** | |  |  | | |  | |  | |  |  |  |
|  | | **Estimate** | **Standard error** | | | **Wald*-Z*** | | **P** | |  |  |  |
| Year‡ | |  |  | | |  | |  | |  |  |  |

| **Female plumage coloration** |  | | |  |  | |  | |  | |  |  | |  |  |
| --- | --- | --- | --- | --- | --- | --- | --- | --- | --- | --- | --- | --- | --- | --- | --- |
| **(N=64)** |  |  |  |  |  |  |  |  |  |  |  |  |  |  |  |
|  | **Estimate** | | | **Standard error** | **DF** | | ***F*** | | **P** | | **Lower** | **Upper** | |  |  |
| **Fixed Effect** |  |  |  |  |  |  |  |  |  |  | **95%** | **95%** | |  |  |
|  |  |  |  |  |  |  |  |  |  |  | **CL** | **CL** | |  |  |
| Intercept | 0.44 | | | 0.53 | 1,39.89 | | 0.85 | | 0.401 | | -0.62 | 1.51 | |  |  |
| Brood size | -0.08 | | | 0.13 | 1,51.27 | | 0.45 | | 0.506 | | -0.35 | 0.17 | |  |  |
| Trophic quality | -0.17 | | | 0.16 | 1,59.77 | | 1.18 | | 0.28 | | -0.48 | 0.14 | |  |  |
| **Random terms** | |  |  | | |  | |  | |  | | |  | |  |
|  | | **Estimate** | **Standard error** | | | **Wald*-Z*** | | **P** | |  |  |  |  |  |  |
| Year | | 0.02 | 0.08 | | | 0.26 | | 0.39 | |  |  |  |  |  |  |

‡After controlling for the fixed effects, there isn't any variation for the year random effect. We thus remove it from the definitive model.

**Table 7 Appendix.** Results of the Linear Mixed Models analysing variation in body mass at fledging and PHA response (normal error structure). Significant terms (95% confidence interval does not include the null) are highlighted in bold and predictors were standardized to improve interpretability. Degrees of freedom are obtained using the Satterthwaite method.

| **Body mass at fledging** |  | | |  |  | |  | |  | |  |  |
| --- | --- | --- | --- | --- | --- | --- | --- | --- | --- | --- | --- | --- |
| **(N=138 owlets in 57 nests)** |  |  |  |  |  |  |  |  |  |  |  |  |
|  | **Estimate** | | | **Standard error** | **DF** | | ***F*** | | **P** | | **Lower** | **Upper** |
| **Fixed Effect** |  |  |  |  |  |  |  |  |  |  | **95%** | **95%** |
|  |  |  |  |  |  |  |  |  |  |  | **CL** | **CL** |
| Intercept | 66.03 | | | 5.94 | 1,50.69 | | 11.11 | | <.0001 | | 54.09 | 77.97 |
| Brood size | -1.43 | | | 1.41 | 1,50.26 | | 1.03 | | 0.316 | | -4.25 | 1.40 |
| Trophic quality | -0.36 | | | 1.48 | 1,33.99 | | 0.06 | | 0.809 | | -3.38 | 2.65 |
| Mother coloration | 0.45 | | | 1.28 | 1,38.15 | | 0.13 | | 0.725 | | -2.13 | 3.04 |
| Father coloration | -0.06 | | | 1.48 | 1,35.08 | | 0.00 | | 0.966 | | -3.06 | 2.94 |
| **Diet diversity** | **7.91** | | | **3.22** | **1,6.57** | | **6.02** | | **0.046** | | **0.18** | **15.64** |
| **Random terms** | |  |  | | |  | |  | |  |  |  |
|  | | **Estimate** | **Standard error** | | | **Wald*-Z*** | | **P** | |  |  |  |
| Year | | 0.68 | 7.97 | | | 0.09 | | 0.46 | |  |  |  |
| Nest_ID | | 29.49 | 23.85 | | | 1.24 | | 0.11 | |  |  |  |

| **PHA response** |  | | |  |  | |  | |  | |  |  | |  |  |
| --- | --- | --- | --- | --- | --- | --- | --- | --- | --- | --- | --- | --- | --- | --- | --- |
| **(N=139 owlets in 57 nests)** |  |  |  |  |  |  |  |  |  |  |  |  |  |  |  |
|  | **Estimate** | | | **Standard error** | **DF** | | ***F*** | | **P** | | **Lower** | **Upper** | |  |  |
| **Fixed Effect** |  |  |  |  |  |  |  |  |  |  | **95%** | **95%** | |  |  |
|  |  |  |  |  |  |  |  |  |  |  | **CL** | **CL** | |  |  |
| Intercept | 0.23 | | | 0.17 | 1,10.24 | | 1.34 | | 0.211 | | -0.15 | 0.61 | |  |  |
| **Owlet body mass** | **0.00** | | | **0.00** | **1,90.43** | | **5.33** | | **0.023** | | **0.00** | **0.01** | |  |  |
| Brood size | -0.03 | | | 0.02 | 1,56.98 | | 2.83 | | 0.098 | | -0.07 | 0.01 | |  |  |
| Trophic quality | 0.00 | | | 0.02 | 1,34.76 | | 0.03 | | 0.855 | | -0.04 | 0.05 | |  |  |
| Mother coloration | 0.00 | | | 0.02 | 1,38.92 | | 0.04 | | 0.842 | | -0.04 | 0.04 | |  |  |
| **Father coloration** | **0.08** | | | **0.02** | **1,38.13** | | **10.6** | | **0.002** | | **0.03** | **0.12** | |  |  |
| Diet diversity | 0.08 | | | 0.07 | 1,37.53 | | 1.47 | | 0.233 | | -0.05 | 0.22 | |  |  |
| **Random terms** | |  |  | | |  | |  | |  | | |  | |  |
|  | | **Estimate** | **Standard error** | | | **Wald*-Z*** | | **P** | |  |  |  |  |  |  |
| Year | | 0.06 | 0.05 | | | 1.18 | | 0.12 | |  |  |  |  |  |  |
| **Nest_ID** | | **0.01** | **0.004** | | | **2.97** | | **0.001** | |  |  |  |  |  |  |

†Reference level

**Figure S1 appendix.** **Correlations among features of trophic quality**. Relationships between the abundance of grasshoppers and insect abundance (A: *r*_p_=0.83, *P* <0.0001) and richness (B: *r*_p_=0.57, *P* <0.0001) (log-transformed) across 542 transects performed in 156 potential scops owl territories. Relationships between the abundance of grasshoppers and insect abundance (C: *r*_p_=0.90,*P*<0.0001) and richness (D: r_p_=0.89, P<0.0001) (log-transformed) across scops owl breeding territories (N= 97 territories). Number of territories is larger than the number of nests where we could study the diet (n=68 nests) because a portion of breeding attempts failed before we could video-film prey delivered in the nests.


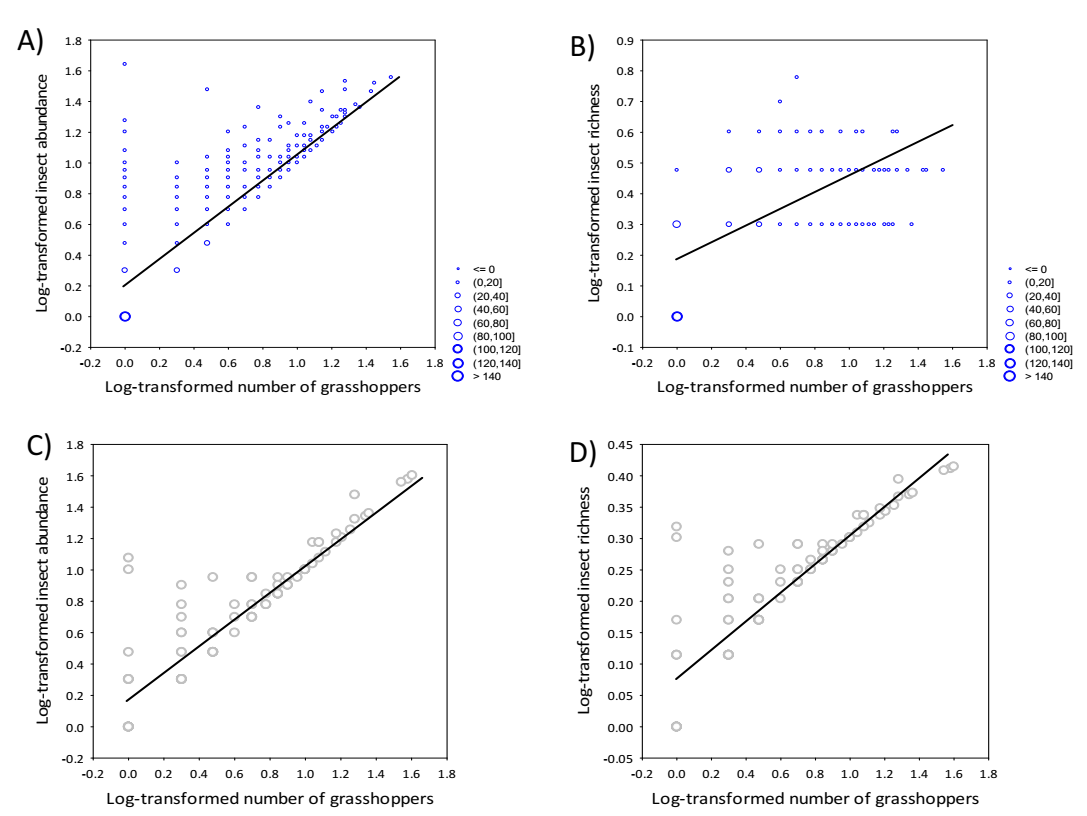


**Figure S2 appendix. Sexual differences in prey delivery in scops owl nests**. Prey brought by fathers had a higher diversity (A), represented a higher biomass (B) and provided proportionally more *Acrididae* prey (C) compared to that of mothers (N=68 nests).

A)

B)

C)

**Figure S3 Appendix. Owlets’ quality in relation to father coloration.** Predicted PHA responses of owlets in relation to father coloration in scops owl nests. Data points are predicted values for owlets from the model on PHA response in the Table 6 of the appendix. The bold-black lines show the best polynomic adjustment of the singular relationship between PHA response and father colour, and the 95% CLs are represented by corresponding dashed lines. N= 139 owlets in 57 nests.


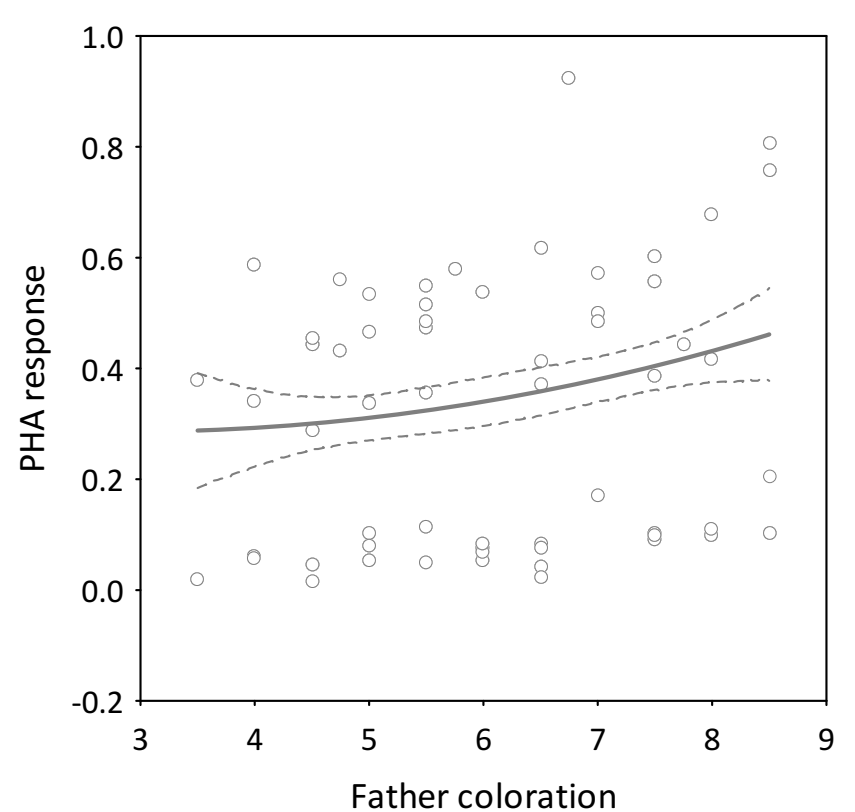


Clarke, A., P. A. Prince, and R. Clarke. 1996. The energy content of dragonflies (Odonata) in relation to predation by falcons. Bird Study **43**:300-304.

Cramp, S. 1998. **Cramp's the complete birds of the Western palearctic.** Optimedia,Oxford University Press, **Oxford**.

Daniel, O., L. Kohli, and M. Bieri. 1996. Weight gain and weight loss of the earthworm Lumbricus terrestris L. at different temperatures and body weights. Soil Biology and Biochemistry **28**:1235-1240.

Fargallo, J. A., J. Navarro-Lopez, P. Palma-Granados, and R. M. Nieto. 2020. Foraging strategy of a carnivorous-insectivorous raptor species based on prey size, capturability and nutritional components. Scientific Reports **10**.

Franco, A., and J. Andrada. 1977. Alimentación y selección de presa en Falco naumanni. Ardeola **23**:137-187.

Frantsevich, L., and H. Cruse. 1997. The stick insect, Obrimus asperrimus (Phasmida, Bacillidae) walking on different surfaces. Journal of Insect Physiology **43**:447-455.

Marchesi, L., and F. Sergio. 2005. Distribution, density, diet and productivity of the Scops Owl Otus scops in the Italian Alps. Ibis **147**:176-187.

Moya-Larano, J., J. M. Orta-Ocana, J. A. Barrientos, C. Bach, and D. H. Wise. 2002. Territoriality in a cannibalistic burrowing wolf spider. Ecology **83**:356-361.

Naef-Daenzer, B., and L. F. Keller. 1999. The foraging performance of great and blue tits (Parus major and P-caerulens) in relation to caterpillar development, and its consequences for nestling growth and fledging weight. Journal of Animal Ecology **68**:708-718.

Zamora-Camacho, F. J., S. Reguera, M. V. Rubino-Hispan, and G. Moreno-Rueda. 2014. Effects of Limb Length, Body Mass, Gender, Gravidity, and Elevation on Escape Speed in the Lizard Psammodromus algirus. Evolutionary Biology **41**:509-517.

Zuffi, M. A., R. Sacchi, F. Pupin, and T. Cencetti. 2011. Sexual size and shape dimorphism in the Moorish gecko (Tarentola mauritanica, Gekkota, Phyllodactylidae). North-Western Journal of Zoology **7**:189-197.
